# In Situ Particle Measurements Deemphasize the Role of Size in Governing the Sinking Velocity of Marine Particles

**DOI:** 10.1029/2022GL099563

**Published:** 2022-11-02

**Authors:** J. R. Williams, S. L. C. Giering

**Affiliations:** ^1^ National Oceanography Centre Southampton UK

**Keywords:** biological carbon pump, size spectra, in situ, sinking velocity

## Abstract

Sinking particles are important in delivering carbon to the deep ocean where it may be stored out of contact with the atmosphere. Whilst particle sinking velocities are known to be influenced by a multitude of factors, size‐based parameterizations remain common in biogeochemical models and in the methods used to determine particulate fluxes from autonomous platforms. Here we carried out an extensive literature review (62 data sets) into the size‐sinking velocity relationship, and find the relationship is much weaker for studies examining particles in situ (median *R* ^2^ = 0.09) compared with ex situ studies (median *R* ^2^ = 0.35). This discrepancy may be because particles examined in the laboratory have more uniform properties than those studied in situ. Our review highlights the shortcomings of using a simple relationship between size and sinking velocity to calculate sinking particulate fluxes in the ocean; considering additional particle characteristics will enable more accurate calculations of particulate fluxes.

## Key Points

- Ex situ studies typically find size to be a strong predictor of sinking velocity; strong correlations are rarely observed in situ however
- Increased homogeneity of other particle characteristics ex situ (e.g., density, composition) are responsible for this discrepancy
- Results suggest importance of these other factors when predicting particle sinking velocities and fluxes using size‐scaling relationships

## 1 Introduction

In the ocean, the production, transfer to depth, and remineralization of organic particles provide a major pathway for the export of carbon from the ocean's surface to the ocean interior (Volk & Hoffert, 1985). Collectively termed the biological carbon pump (BCP), these processes act to maintain atmospheric CO_2_ approximately 200 ppm lower than they would otherwise be (Maier‐Reimer et al., 1996; Parekh et al., 2006). Although several processes contribute to the BCP, the gravitational settling of organic particles are thought to result in ∼1,000 Pg of ocean carbon storage (Boyd et al., 2019), up to 90% of the carbon sequestered by the BCP (Boyd et al., 2019; Buesseler et al., 2020; Sarmiento & Gruber, 2006).

**Figure 1:**
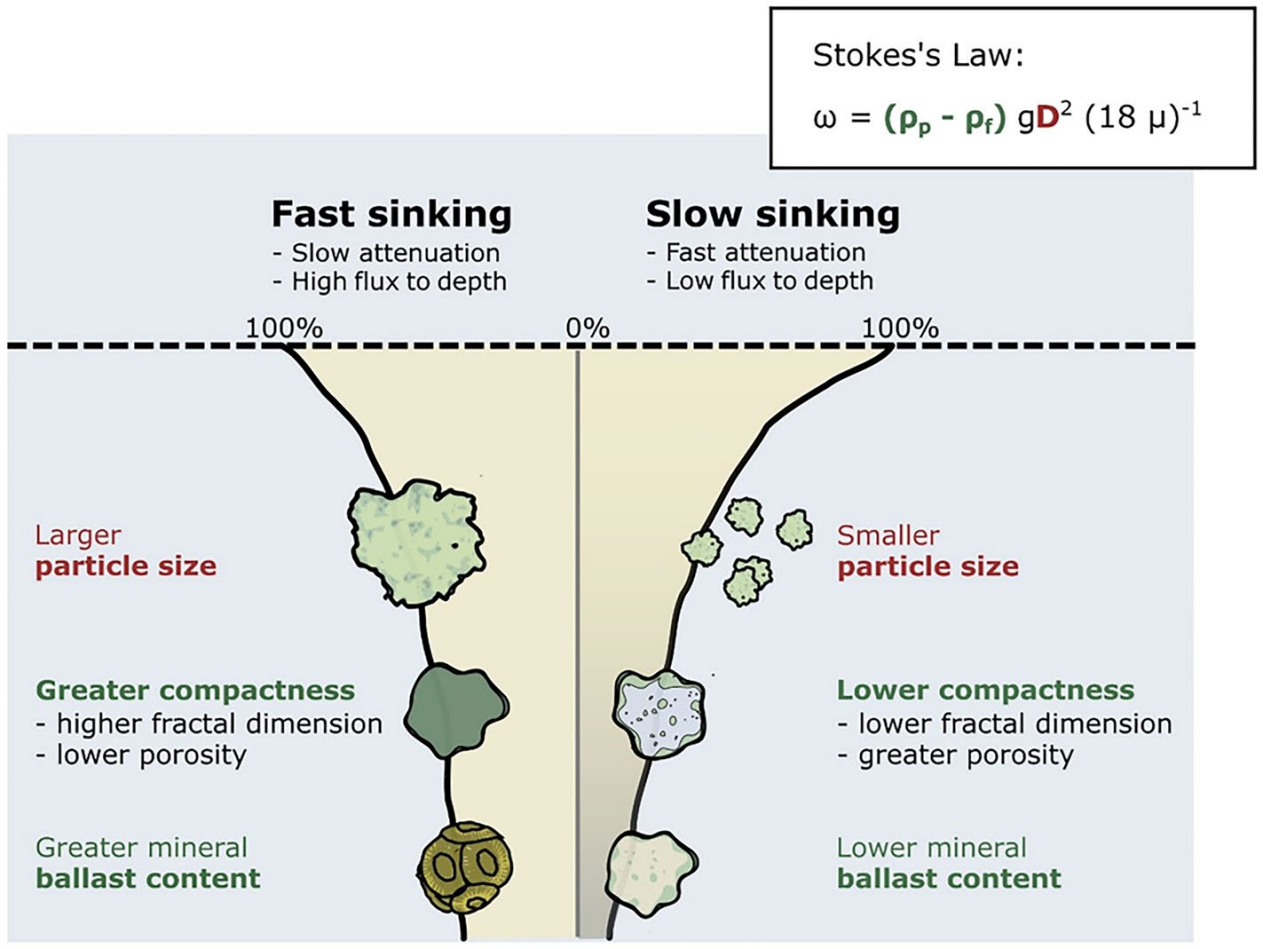
Schematic illustrating factors posed to influence particle sinking velocity, their relation to size (red) or density (green) as described in Stokes's law, and the effect of fast and slow sinking particles on particulate flux attenuation rates and particles fluxes reaching depth. Illustrative flux attenuation curves are shown for fast‐sinking (left) and slow‐sinking (right) particles. Fast sinking particles experience slower rates of flux attenuation due to decreased duration of exposure to remineralization whilst they sink.

As particulate organic carbon (POC) sinks, proportions of this downward flux are reworked by metazoans such as zooplankton, and eventually remineralized back into CO_2_, through both microbial and zooplankton respiration (Giering et al., 2014; Steinberg et al., 2008; van der Jagt et al., 2020). As a result of this particle remineralization and reworking, sinking POC fluxes are observed decrease with depth. The rate of flux attenuation (and hence the proportion of sinking carbon reaching the deep ocean) is determined by the balance between particle sinking velocities and remineralization rates (Bach et al., 2019; Marsay et al., 2015). Since particle sinking velocities determine the length of time in which a particle is exposed to metazoan and microbial remineralization, sinking velocity is a crucial determinant in the degree of attenuation of POC fluxes and BCP efficiency (Laurenceau‐Cornec et al., 2015) (Figure 1).

In recent years, the use of in situ optical methods has emerged as an important tool in the study of the BCP (Giering, Cavan, et al., 2020). Increasingly able to be deployed autonomously (Lombard et al., 2019; Picheral et al., 2022), these methods can provide far greater spatiotemporal resolution and coverage than traditional ship‐based sampling methods (Giering, Cavan, et al., 2020; Lombard et al., 2019). Given also the considerable effort that has focused on improving the utility of in situ imaging devices, in situ cameras are now capable of providing quantitative particle information on particles from 1 to 10,000 μm in diameter (Lombard et al., 2019). Using the particle size distributions obtained by in situ imaging methods, particle fluxes within a given size class can be calculated if sinking velocities of particles within the size class can also be estimated (McDonnell & Buesseler, 2010, 2012), or, more commonly, through directly relating particle size to flux via an empirical relationship (Guidi et al., 2008; Iversen et al., 2010). A robust understanding of the factors that govern particle sinking rate is therefore crucial in the implementation of these cutting‐edge methods for estimating particulate fluxes and studying the BCP.

Whilst the use of particle size spectra provide the means to calculate particulate fluxes with unprecedented resolution, the prevailing methods used to empirically relate size to fluxes assume that mass and sinking velocity as functions of particle size remain constant (Guidi et al., 2008; Iversen et al., 2010). Further, relying on a single relationship to describe sinking velocity in terms of particle size implies that larger particles should always sink faster than smaller ones (McDonnell & Buesseler, 2010). Although several studies have observed size to exert a strong control on sinking velocities (Alldredge & Gotschalk, 1988; Iversen et al., 2010), considering size as a key predictor of sinking velocity assumes relative constancy of other particle properties such as particle composition, porosity and biomineral content. In recent years, empirical studies have indicated this assumption of constancy can not be applied in situ (Iversen & Ploug, 2010; Laurenceau‐Cornec et al., 2020; Ploug et al., 2008) and that size alone is often a poor predictor of sinking velocity (Diercks & Asper, 1997; Iversen & Lampitt, 2020). Nevertheless, size‐based methods remain a commonly used approach to estimate fluxes from in situ image data (Cram et al., 2022; Fender et al., 2019; Guidi et al., 2016; Kiko et al., 2017, 2020; Ramondenc et al., 2016), and size‐based parameterizations of vertical carbon fluxes remain common in biogeochemical models (Aumount et al., 2015; Kriest & Oschlies, 2008; Leung et al., 2021; Swart et al., 2019; Yool et al., 2021).

Here we first outline the theoretical basis underpinning commonly used size‐based approaches. We then highlight the lack of evidence suggesting size can represent a strong predictor of marine particle sinking velocities in situ, and assess the reasons for differences between studies. We recommend avenues for further study that will facilitate improved mechanistic understanding of particle sinking velocities and broaden the applicability of in situ image‐based estimations of particle flux.

### 1.1 The Theoretical Basis for Size‐Based Methods

In recent decades, derivations such as Stokes's law have been widely used to estimate particle sinking velocity (Laurenceau‐Cornec et al., 2020). Assuming that particle drag coefficients can be calculated as a simple function of Reynolds number for low Reynolds numbers (in laminar flow conditions), and balancing drag and gravitational forces on a particle, these derivations pose size to be a key determinant of sinking velocity. Stokes's Law says that

(1) $$w=\left(\rho_{p}-\rho_{f}\right)\frac{gD^{2}}{18\mu }$$

where *w* is the sinking velocity of a sphere (m s^−1^), *ρ*_*p*_ and *ρ*_*f*_ are the sphere and fluid densities (kg m^−3^), *g* is the acceleration due to gravity (9.81 m s^−2^), *D* is the sphere diameter (m),and *μ* is the fluid dynamic viscosity in kg m^−1^s^−1^. Power law functions based on Stokes's Law have been used to relate particle size to sinking velocity for decades (Alldredge & Gotschalk, 1988; Smayda, 1970) and more recently to parameterize modeled particle sinking velocities (Aumont et al., 2015; DeVries et al., 2014; Kriest & Evans, 1999; Leung et al., 2021). Other biogeochemical models simply incorporate size through discrete size classes, with a large, fast‐sinking fraction, and a small, slow‐sinking fraction (Aumont et al., 2015; Swart et al., 2019; Yool et al., 2021). The lack of mechanistic understanding as to how well size constrains sinking velocity has resulted in a variety of size‐sinking relationships in Earth system models, which yield up to order of magnitude differences in sinking velocity for particles of the same size, and introduce uncertainty into flux prediction and biogeochemical models (Cael et al., 2021; Niemeyer et al., 2019). Since plankton models additionally suggest a decrease in cell size with warming, constraining sinking velocities in an accurate mechanistic fashion is of importance for the accurate modeling of climate change projections (Cael et al., 2021; Finkel et al., 2010).

Power law functions have more recently also been used to directly estimate particulate fluxes from particle size distributions. Since both sinking velocity (*w*) and particle mass (*m*) and hence flux for a given particle *i* (*F*_*i*_) can be expressed as power law functions of the form (*y = ax* ^*b*^), their product can be expressed in the same form:

(2) $$F_{i}=wm=AD^{B}$$

where *D* is particle diameter, and *A* and *B* are constants (Guidi et al., 2008). If *A* and *B* are known, size spectra can be used to calculate total mass fluxes, *F*. *A* and *B* may be estimated through a minimization procedure (Cram et al., 2022; Fender et al., 2019; Guidi et al., 2008; Iversen et al., 2010; Nowald et al., 2015) if alternative measurements of particulate fluxes can be made, and assuming that mass and particle size as a function of depth are constant for all depths (Iversen et al., 2010). Alternatively, when additional flux measurements have not been made (such as on autonomous deployments on moorings, gliders, or floats), prior studies can be used to estimate global values for *A* and *B* (Guidi et al., 2008, 2016; Kiko et al., 2020; Ramondenc et al., 2016). The above approaches assume that particle mass and sinking velocity as functions of size are constant with depth and, in the latter instance, universally constant; hence both methods represent size as a strong control of sinking velocity.

### 1.2 Empirical Evidence on the Size‐Sinking Velocity Relationship

To direct our discussion in the most constructive fashion toward particle characteristics commonly discussed in the literature, we focused our analysis on four of the most commonly studied characteristics. It should however be noted that the frequency with which characteristics are discussed in the literature does not necessarily indicate that they are the most important four drivers of sinking velocity. To identify these characteristics, we carried out a literature search into studies measuring particle sinking velocity and associated particle characteristics using both in situ and ex situ methods, and commonly used keyterms to describe marine particle characteristics (“Size,” “Ballast,” “Morphology,” “Composition,” “Type,” “Shape,” “Compactness,” “Fractal” [Dimension]). Restricting results to within Earth and Planetary Sciences, we searched for abstracts, titles, and keywords containing the words “Particle” and “Sinking” and “Velocity” as well as a given particle characteristic. Size returned the greatest number of studies (79), followed by parameters relating to chemical and taxonomic composition (Composition: 37; Type: 22; Ballast: 18). Searches relating to other morphological properties typically returned the fewest results (“Shape”: 17; “Fractal” [Dimension]: 5; “Compactness”: 1; “Permeability”: 1; “Morphology”: 1).

**Figure 2:**
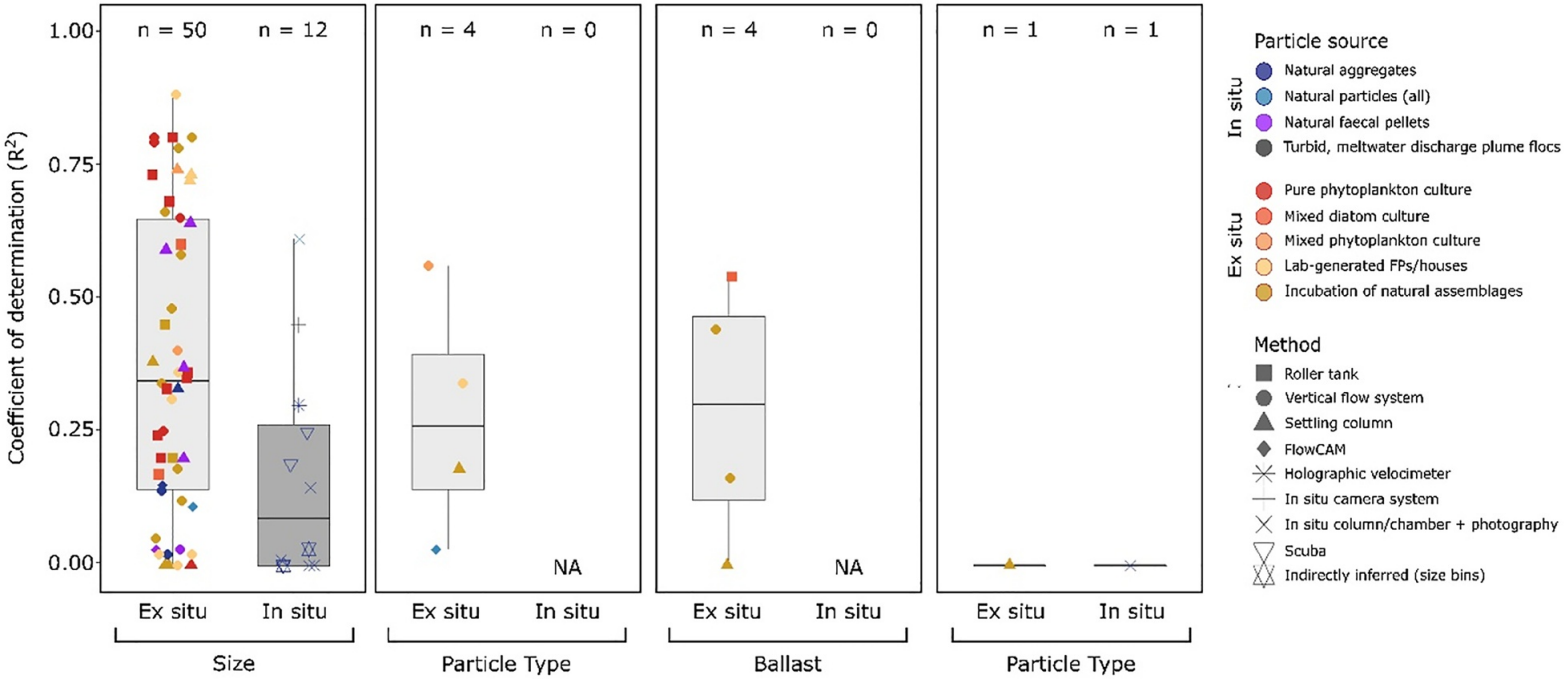
Boxplot comparing proportion of variance in sinking velocity explained by particle characteristics. Coefficients of determination (*R* ^2^) from linear models and analyses of variance performed between particle characteristics (size, type, ballast content, shape) and sinking velocities directly measured in previous studies (see text, Table S1 in Supporting Information S1). Colors of boxplots indicate whether sinking velocity measurements were made in situ (dark gray) or ex situ (light gray), and how particles were generated (in situ: natural particles observed in situ or measured immediately ex situ without prior incubation; ex situ: cultured or incubated ex situ prior to measurement. Shapes of the points indicate method used to measure sinking velocity.

Using the four most commonly studied particle attributes from our literature search (size, particle type, ballast, and shape), we identified 62 data set from 38 studies and examined the degree of correlation between sinking velocity and each of the above attributes (Figure 2). A full description of methods is provided in Section 3. Briefly, for particle type, ballast, and shape, *R* ^2^ (proportion of variance in sinking velocity explained by size) were calculated from linear regressions for continuous variables, or from analyses of variance (ANOVAs) for categorical variables. To assess the degree of variation in sinking velocity explained by particle size in each study, a power law function was fitted to the data, and *R* ^2^ of this power law function calculated using a linear regression on the log‐log plot of size against sinking velocity. A power law funtion was chosen over a linear relationship since sinking velocity is thought to scale with particle diameter according to a power law function according to Stokes's Law and empirically modified versions incorporating porosity (Guidi et al., 2008; Laurenceau‐Cornec et al., 2020; Xiang et al., 2022).

Despite particle size having received the most interest, our review suggests the dependency of sinking velocity on size is not well constrained (Figure 2). Size explains between 0% and 88% in the variation in particle sinking velocity (as determined by the coefficient of determination “*R* ^2^”) with a median value of 31%. The strongest correlation between size and sinking velocity was observed for intact salp fecal pellets from the Southern Ocean (Iversen et al., 2017). However, in 26% of the data sets, particle size was observed to be a poor predictor of sinking velocity, explaining less than 10% of variation in particle sinking velocity (Figure 2). Particle size did not appear to be a stronger predictor of sinking velocity than particle type or particle ballast content (Wilcoxon rank tests, *p* > 0.8). The median percentage of variance in sinking velocity explained by particle type and ballast content were 26% and 30%, respectively. It is noteworthy however that only four ex situ data sets examined the influence of particle type (i.e., differences in both taxonomic composition, i.e., aggregates made of different phytoplankton species, or particle shape, e.g., fecal pellets vs. aggregates) or ballast content. Likewise for particle shape, only one ex situ (Laurenceau‐Cornec et al., 2015) and one in situ (Iversen & Lampitt, 2020) study directly measured a particle shape characteristic (aspect ratio) and sinking velocity, with neither of these studies finding sinking velocity to be explained by particle shape alone.

**Figure 3:**
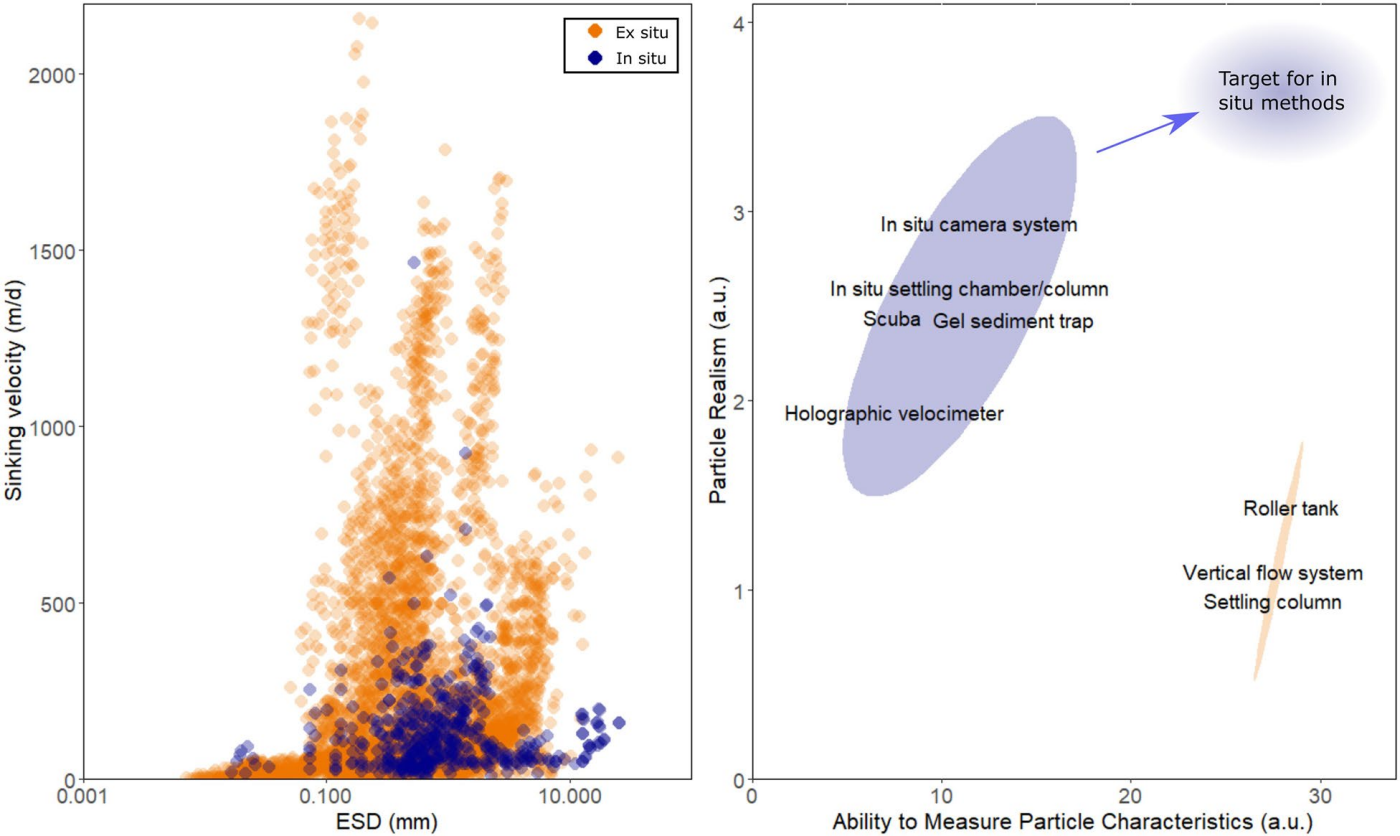
(a) Particle size (equivalent spherical diameter [ESD]) against sinking velocity for all particles in this study, from both in situ (blue) and ex situ (orange) studies. (b) Relative advantages of in situ and ex situ methods (blue and orange respectively) in terms of ability to measure particle characteristics and particle realism, when investigating the relationship between particle sinking velocity and particle characteristics. Position of each method relates to an assigned “Particle Realism score” and “Measurement Capability score” as described in Section 3.3; explanations of assertions used for scoring these methods available in Table S2 in Supporting Information S1.

For the data sets focusing on particle size as a predictor, we found strong differences between measurements made in situ and ex situ. *R* ^2^ values were significantly higher for ex situ studies than in situ studies (Wilcoxon rank test, *p* < 0.05), suggesting that the strength of the size‐sinking velocity relationship may be influenced by whether measurements are made in or ex situ. While weak correlations between size and sinking velocity were observed in both situ and ex situ data sets, strong dependencies of sinking velocity on particle size were only observed ex situ. However, when combining all ex situ data sets, a clear lack of a “global” size‐to‐sinking velocity relationship becomes apparent (*n* = 4,138, *p* = <0.001, *R* ^2^ = 0.193); Figure 3a; though note that these studies used different method, which may forego a direct comparison (Giering, Hosking, et al., 2020). For in situ data sets (*n* = 12), size explained less than 30% of variability in sinking velocity in all but two studies which respectively examined flocs from meltwater discharge plumes and resuspended near‐bottom sediment. For in situ particles, the median percentage of variance in sinking velocity explained by particle size was 9%, contrasting with 35% for particles measured ex situ. Overall these findings suggest that the strong relationships observed ex situ between individual particle characteristics and sinking velocity rarely hold true in situ. As such, the methodological biases outlined below should be taken into consideration before extrapolating relationships observed in ex situ studies to natural marine particles in situ.

### 1.3 Ex Situ Versus in Situ: Methodological Compromises

Methodological differences between in situ and ex situ studies can explain both the predominance of ex situ studies examining the size‐sinking velocity relationship, and why size is seen to more tightly constrain sinking velocities ex situ than in situ.

In situ methods hold the major advantage of observing particles in their natural environment. Any measurements made are therefore acquired without the need for handling particles, decreasing (but not eliminating [Briggs et al., 2011; Cetinić et al., 2012]) the potential alteration and disturbance to particle properties (Giering, Cavan, et al., 2020; Iversen & Lampitt, 2020), and thus maximizing the realism of any interactions between sinking velocity and particle characteristics. However, a major drawback of in situ optical devices is that these methods lack the capability to provide direct information on a number of particle characteristics, such as particle density and composition (Giering, Hosking, et al., 2020). These methods must hence rely on additional data or assumptions to estimate particle sinking velocities and calculate particulate fluxes. Given these uncertainties, the expensive nature of in situ camera systems, and a lack of standardization in analysis routines for in situ image data sets (Giering, Hosking, et al., 2020), in situ studies into particle sinking velocities remain sparse compared with more traditional ex situ methods. In summary, in situ studies lack the capacity to study particle characteristics which may be measured ex situ, but maximize realism (Figure 3b).

Most studies into factors constraining sinking velocity involve incubating particles ex situ prior to or during measurements. By examining particles in a laboratory, detailed measurements of a wide number of particle characteristics can be made, such as chemical and taxonomic composition, removing the need for estimates of these parameters (Mantovanelli & Ridd, 2006). In addition, studies where particles are generated ex situ also allow for manipulation of particles characteristics, to test for effects of specific particle characteristics on sinking velocities (Giering, Cavan, et al., 2020). However, ex situ particles are likely not reflective of in situ particle dynamics, partly owing to ex situ particles being more homogenous. Three aspects contribute to this discrepancy: (a) Homogeneity in the “source” particle pool; (b) homogenization of particles during particle collection; and (c) homogenization of particles during the incubation for measuring sinking velocities.

Firstly, particles for ex situ incubations are often sourced from “artificial,” laboratory‐produced particles, for example, incubating homogenous particle pools such as phytoplankton cultures in roller tanks, whilst in the natural environment a heterogenous pool of particles of varied age, composition, density, structure, and porosity exists (Alldredge, 1998; Alldredge & Gotschalk, 1988; Iversen & Lampitt, 2020). The unnatural homogeneity of ex situ particles sourced in this way reduces the variability in sinking velocity introduced by factors other than size, thus allowing size to exert a dominant control over sinking velocity.

An alternative approach to sourcing particles for incubations involves the collection of natural marine particles. Whilst this approach allows for collection of a more diverse particle pool, highly fragile marine aggregates are susceptible to damage, alteration, and compaction or disaggregation during sampling for ex situ incubations (Alldredge & Gotschalk, 1988; Alldredge & Silver, 1988; Giering, Cavan, et al., 2020; Iversen & Lampitt, 2020; Kajihara, 1971; Takeuchi et al., 2019). Particles measured in the laboratory are able to withstand higher turbulences than that observed in the ocean, (Alldredge et al., 1990; Riebesell, 1992), and also typically exhibit increased sinking velocities compared with measurements made in situ (Figure 3a) (Alldredge & Gotschalk, 1988; Shanks & Trent, 1980). These observations indicate that, despite the more heterogenous nature of naturally occurring particles compared to cultured particles, sampling in this way has a tendency to alter particles (and/or particle populations) such that they are no longer fully representative of marine particles in situ.

Lastly, particles incubated ex situ are exposed to a far more limited set of processes influencing their formation and composition. In situ, a number of biological and physical processes contribute to the aggregation of particles. For physical processes, mechanisms such as Brownian motion, differential settling (in which faster sinking particles scavenge slower sinking or suspended particles upon collision), and turbulent shear (McCave, 1984) influence aggregate formation, with the importance of these processes varying depending on particle size (Jackson, 1994; McCave, 1984; Takeuchi et al., 2019). By contrast, in roller tanks turbulent shear is negligible in aggregate formation once the initial spin‐up period is over (Engel et al., 2009; Laurenceau‐Cornec et al., 2015), whilst in Couette chambers the influence of shear is amplified relative to in situ (Jackson, 2015; Lick et al., 1993). Ex situ incubations also typically lack the diversity of biologically mediated processes that aggregate or disaggregate particles. In situ, aggregation processes in situ can include compaction into fecal pellets, the accretion of particles onto mucous houses and other exuded exopolymers (Hamner et al., 1975; Hansen et al., 1996; Kiørboe, 2001), and aggregation due to organisms' feeding currents (Fukuda & Koide, 2000). Particle disaggregation as a result of zooplankton feeding can also occur in situ, a process which may not be included in ex situ incubations (Dilling & Alldredge, 2000; Iversen & Poulsen, 2007). Given that in situ studies cannot replicate the diversity of in situ processes involved in forming and transforming particles, it is unsurprising that ex situ particle pools are more homogenous that those in situ, and hence exhibit stronger size‐scaling relationships. Overall, ex situ studies favor the ability to measure and examine particle dynamics in detail, whilst sacrificing realism (Figure 3b).

## 2 An Outlook for the Use of In Situ Methods

This review highlights the discrepancy in the extent to which size controls sinking velocity between in situ and ex situ studies (Alldredge & Gotschalk, 1988), and that—despite this discrepancy—size‐based methods remain common for estimating fluxes from in situ data or representing marine particle particles in models.

While there is clear merit in using simple size‐to‐sinking velocity relationships for autonomous methods (e.g., Fender et al., 2019; Guidi et al., 2008; Iversen et al., 2010), these approaches provide limited mechanistic understanding into the size‐flux relationship, limiting the certainty with which relationships can be spatiotemporally extrapolated. As suggested by McDonnell and Buesseler (2010), taking into account particle types will increase the range of spatiotemporal scales over which size‐scaling relationships can be applied. Recognizing individual size‐scaling relationships for varying particle types will enable more accurate sinking velocity and flux estimates for each particle type. In turn, considering the weighted contribution of each particle type will maintain the accuracy of optical methods even under varied ecological and biogeochemical settings, when contributions from each particle type may vary. Alternatively, the inclusion of additional particle characteristics, such as compactness or bulk particle composition, into a unified equation (e.g., Giering, Hosking, et al., 2020) may provide more accurate predictions of particle sinking velocities. Some information of these characteristics can be obtained from optical measurements, such as porosity (Bach et al., 2019), bulk density (Hurley et al., 2016; Neukermans et al., 2012), and bulk particle composition (inorganic/organic ratios, Loisel et al., 2007; Twardowski et al., 2001).

Yet, at present, the uncertainties associated with these proxies are large or unconstrained. Simultaneous measurements of particle type (or characteristics), size and sinking velocity will enable the development of these methods, and is likely to be expedited by advances in machine learning (Giering, Cavan, et al., 2020; Iversen & Lampitt, 2020). Moving away from purely size‐based velocity and flux relationships to incorporate these additional particle properties will not only facilitate improved mechanistic understanding of particle sinking and the BCP, but also promote increased spatio‐temporal resolution of methods used to the study the BCP, through the use of autonomous platforms and in biogeochemical models.

## 3 Methods

### 3.1 Data Compilation

We compiled observations of particle sinking velocity and associated particle characteristics from 62 data sets from 38 studies (see Table S1 in Supporting Information S1). These data had previously been compiled by Cael et al. (2021) and Laurenceau‐Cornec et al. (2015, 2020); all original data sets were validated and, if needed, redigitized using Plot Digitizer (https://automeris.io/WebPlotDigitizer/). Studies not relating to marine particles were excluded from this analysis. In the small number of cases where particle size and sinking velocity data had been fitted to a power law function in original studies (*n* = 10), published *R* ^2^ values in the literature were used. Data were assigned to “in situ” and “ex situ” groups for measurement type, based on the method used to measure sinking velocity in each study. The particle types examined in each study were assigned to one of nine particle types (e.g., natural aggregates, mixed diatom culture; for full list Figure 2, Table S1 in Supporting Information S1), with method used to measure particle sinking velocities also described through one of nine groups (e.g., Scuba photography, Vertical flow system; for full list, see legend of Figure 2, Table S1 in Supporting Information S1).

### 3.2 Sinking Velocity/Particle Characteristic Analyses

To assess the variability in sinking velocity explained by a particle size in each study, a power law function (in form *w = Ad* ^*B*^, where *w* is the sinking velocity, *d* the diameter, and *A* and *B* are scaling coefficients) was fitted to the data.

For particle type, ballast, and shape, *R* ^2^ were recorded either from performing linear regressions or ANOVAs, depending on whether the particle characteristic was described in terms of continuous or categorical data. For example, in some studies particle type was analyzed as a categorical variable with discrete groups such as *S*. *costatum* or *E*. *huxleyi* aggregates, and sinking velocity was compared between these groups by means of an ANOVA. In another study, particle type was expressed as a percentage of aggregate composition of one diatom morphotype (Laurenceau‐Cornec et al., 2015). In this case, a linear regression was performed between percentage of total composition and particle sinking velocity. Lastly, having failed both Levene's and Shapiro Wilk tests, a Wilcoxon rank sum test with continuity correction was performed to assess whether *R* ^2^ coefficients differed significantly between in situ and ex situ studies.

### 3.3 Methodological Comparison

To represent the advantages and disadvantages of in situ and ex situ methods for sinking velocity measurement, methods were ranked in terms of their ability to measure particle characteristics, and in terms of particle realism. Although these assertions are subjective rankings, a scoring system was devised to standardize rankings and criteria by which methods were judged. For measurement capability score, particle characteristics (Size, Ballast, Taxonomic composition/Particle type, Chemical composition, Shape, Dry weight, Porosity, Fractal dimension, Density, and Sinking velocity) were assigned a score from 0 to 4, describing the comprehensiveness with which a particle characteristic could be studied with a given method (0 lowest, 4 highest; see Table S2 in Supporting Information S1). Measurement capability scores of individual characteristics were summed to give an overall score. Where a range of measurement score was given for a particle characteristic, the mean value was used when summing scores to calculate (e.g., 2–3 scored as 2.5).

For the particle realism score, each method was assigned a score from 0 to 4, based on the extent to which the particles measured had been influenced by sampling and measurement procedures, that is, the extent to which particle communities measured could be expected to reflect natural marine particle communities in situ. A brief explanation for assigned scores and evidence supporting these assertions are outlined in Table S2 in Supporting Information S1).

## Conflict of Interest

The authors declare no conflicts of interest relevant to this study.

## Supporting information

Supporting Information S1

Table S1

Table S2

Data Set S1

## Data Availability

All data used in this work were accessed through previously published studies and are provided in Data Set S1. Table S1 in Supporting Information S1 provides the data source details, and all studies are cited within our references. Table S2 in Supporting Information S1 provides assertions underpinning schematic in Figure 3b and references underpinning these assertions.
