# Supplementary material for: In Situ Particle Measurements Deemphasize the Role of Size in Governing the Sinking Velocity of Marine Particles
Source: Geophys Res Lett. 2022 Nov 2;49(21):e2022GL099563. doi: 10.1029/2022GL099563 (PMC9786660; doi:10.1029/2022GL099563)
Supplement: Supplementary file 1 — Supporting Information S1 [file GRL-49-e2022GL099563-s001.pdf]

[Geophysical Research Letters]

Supporting Information for

[In situ Particle Measurements Deemphasize the Role of Size in Governing Particle Sinking Velocity]

[J. R. Williams[1], S. L. C. Giering[1]]

[[1]National Oceanography Centre, Southampton, UK]

Contents of this file

Additional Supporting Information (Files uploaded separately)

Caption for Table S1
Caption for Table S2
Caption for Data Set S1

Introduction

These supporting information in the form of xlsx files set out the raw and processed data used in this study, and identifiers to all raw data analysed in this study. Data processing involved fitting a power law function to size and sinking velocity datasets, and fitting either linear regressions or Analyses of Variance (ANOVAs) to particle type, ballast content, and shape datasets. No known imperfections or anomalies have been identified in the data.

Table S1. Outputs from all analyses (power law fits, linear regressions, ANOVAs) for all studies analysed here, along with additional metadata for each study. Studies are identified by a paper number which is referred to identify datasets in Data Set S1. The "All parameters" sheet contains outputs for all analyses; separate sheets for individual parameters are also included to separate out analyses by particle characteristic, for convenience. Following the naming convention: "Williams & Giering_TableS1"

Table S2. Contains the Measurement Capability Scores and Particle Realism Scores assigned to methods of measuring sinking velocity, presented in Figure 3. Also included are explanations of the scoring system and of assertions used to assign each method a score. Following the naming convention: "Williams & Giering_TableS2"

Data Set S1. Contains all raw data used in this study. This data will additionally be made publicly available following revisions and prior to resubmission. "Study number" column is an index to match datasets to studies; these studies and their corresponding index number are outlined in Table S1. A README sheet is included to explain the indexing system. "Williams & Giering_DS01"
